# Supplementary material for: Stigma processes, psychological distress, and attitudes toward seeking treatment among pedohebephilic people
Source: PLoS One. 2024 Oct 24;19(10):e0312382. doi: 10.1371/journal.pone.0312382 (PMC11500907; doi:10.1371/journal.pone.0312382)
Supplement: S1 Table — (DOCX) [file pone.0312382.s001.docx]

**S1 Table. Means, *SD*s and standardized factor loadings of items from the anticipated negative therapist behavior upon disclosure scale (*N* = 286)**

| **Item** |  |  |  | **Standardized factor loadings** | | | | |
| --- | --- | --- | --- | --- | --- | --- | --- | --- |
|  |  |  |  |  | **Second-order model** | | | |
|  | *M* | *SD* | **UM 1** | **UM 2** | **RCR** | **ATB** | **TD** | **GF** |
| **1. respect patient confidentiality (-)** | 4.31 | 2.10 | .77 | - |  |  |  |  |
| **2. treat me with respect (-)** | 4.14 | 2.03 | .82 | .80 | .85 |  |  |  |
| **3. pressure me to do interventions that I do not want to do** | 4.40 | 1.92 | .66 | .65 | .70 |  |  |  |
| **4. stop seeing me** | 3.83 | 1.88 | .75 | - |  |  |  |  |
| **5. report me to the authorities** | 3.90 | 2.03 | .78 | .75 |  | .76 |  |  |
| **6. say hurtful things** | 3.77 | 2.08 | .78 | .80 |  | .82 |  |  |
| **7. tell me that he or she cannot work with me any longer** | 4.17 | 1.85 | .79 | .79 |  |  | .83 |  |
| **8. warn others about me** | 3.70 | 2.01 | .79 | - |  |  |  |  |
| **9. stop trusting me** | 4.23 | 2.10 | .84 | .83 |  | .85 |  |  |
| **10. try to change my sexual interests** | 4.38 | 2.08 | .64 | - |  |  |  |  |
| **11. continue therapy (-)** | 4.45 | 1.62 | .71 | .71 |  |  | .74 |  |
| **12. appreciate me more (-)** | 2.49 | 1.78 | .62 | .60 | .66 |  |  |  |
| **13. reject me** | 4.05 | 1.89 | .82 | .83 |  |  | .88 |  |
| **14. ridicule me** | 2.83 | 1.82 | .69 | .70 |  | .71 |  |  |
| **15. pay close attention to my needs (-)** | 3.75 | 1.85 | .77 | .76 | .80 |  |  |  |
| **16. choose therapeutic interventions that suit my goals and needs (-)** | 3.84 | 1.92 | .77 | - |  |  |  |  |
| **Reduced Collaboration/ Respect (RCR)** |  |  |  |  |  |  |  | .91 |
| **Adversarial Therapist Behavior (ATB)** |  |  |  |  |  |  |  | .98 |
| **Treatment Discontinuation (TD)** |  |  |  |  |  |  |  | .93 |

*Note.* Item stem: “If I disclose a sexual interest in children to a therapist, I would expect that the therapist will...” Items with a (-) were inverted. Items could be rated on a scale ranging from 1 to 7, whereby higher scores reflect a higher level of agreement.

UM1 = unifactorial model, UM2 = 11-item unifactorial model, GF = general factor, RCT = Reduced Collaboration/ Respect, ATB = Adversarial Therapist Behavior, TD = Treatment Discontinuation
