# Supplementary material for: Stigma processes, psychological distress, and attitudes toward seeking treatment among pedohebephilic people
Source: PLoS One. 2024 Oct 24;19(10):e0312382. doi: 10.1371/journal.pone.0312382 (PMC11500907; doi:10.1371/journal.pone.0312382)
Supplement: S2 Table — (DOCX) [file pone.0312382.s002.docx]

**S2 Table. Means, SDs and Standardized Factor Loadings of Items assessing anticipated stigma (from Fear of Rejection/Concealment subscale of the Proximal Stigmas Scale for Minor Attracted People, *N* = 283)**

| **Item** |  |  | **Standardized factor loadings** | |
| --- | --- | --- | --- | --- |
|  | *M* | *SD* | **Model 1** | **Model 2 without item 5** |
| **I would not tell my friends that I am sexually attracted to children because I would be afraid of losing them.** | 5.75 | 1.79 | .71 | .71 |
| **When I like a child, I do anything to prevent people from finding out.** | 5.55 | 1.75 | .79 | .79 |
| **I am careful of what I say to avoid showing that I am a minor attracted person.** | 5.99 | 1.53 | .72 | .72 |
| **When I feel sexually attracted to a child, I hope no one realizes it.** | 6.29 | 1.26 | .79 | .79 |
| **It is difficult for me to say that I am a minor attracted person, including to someone I know.** | 5.90 | 1.76 | .72 | .73 |
| **At school and/or work, I pretend to be interested in adults (e.g., pretending to be attracted to women or men).^a^** | 4.64 | 2.15 | .23 | - |

^a^ This item was deleted because of the low factor loading.
