# Supplementary material for: Stigma processes, psychological distress, and attitudes toward seeking treatment among pedohebephilic people
Source: PLoS One. 2024 Oct 24;19(10):e0312382. doi: 10.1371/journal.pone.0312382 (PMC11500907; doi:10.1371/journal.pone.0312382)
Supplement: S3 Table — (DOCX) [file pone.0312382.s003.docx]

**S3 Table. Means, SDs and standardized factor loadings of items for internalized stigma (from the internalizing symptoms regarding minor-attraction subscale, *N* = 283)**

| **Item** |  |  | **Standardized factor loadings** | |
| --- | --- | --- | --- | --- |
|  | *M* | *SD* | **Model 1** | **Final model without item 7** |
| **Life as an MAP is not as fulfilling as life as a non-MAP.** | 4.91 | 2.06 | .67 | .66 |
| **Whenever I think a lot about being an MAP, I feel critical about myself.** | 3.89 | 2.26 | .79 | .79 |
| **I am confident that my minor attraction does not make me inferior.** | 5.50 | 1.88 | .68 | .69 |
| **Whenever I think about being an MAP, I feel depressed.** | 3.85 | 2.10 | .79 | .78 |
| **Attraction to children is deviant.** | 3.42 | 2.18 | .49 | .49 |
| **Being an MAP is a satisfactory and acceptable way of life for me.** | 4.34 | 1.96 | .82 | .82 |
| **Most MAPs end up lonely and isolated.** | 5.15 | 1.49 | .36 | - |
| **I have no regrets about being an MAP.** | 4.31 | 2.14 | .80 | .81 |
