# Supplementary material for: Stigma processes, psychological distress, and attitudes toward seeking treatment among pedohebephilic people
Source: PLoS One. 2024 Oct 24;19(10):e0312382. doi: 10.1371/journal.pone.0312382 (PMC11500907; doi:10.1371/journal.pone.0312382)
Supplement: S4 Table — (DOCX) [file pone.0312382.s004.docx]

**S4 Table. Means, SDs and Standardized Factor Loadings of Items from the Knowledge about Psychotherapy Scale (*N* = 286).**

| **Item** | ***M*** | ***SD*** | **Standardized factor loadings** |
| --- | --- | --- | --- |
| **I know nothing about what happens in therapy < > I know a lot about what happens in therapy** | 73.87 | 27.62 | .81 |
| **I do not know about my rights as a patient < > I know a lot about my rights as a patient** | 62.82 | 32.44 | .68 |
| **I do not know what to expect from psychotherapy < > I know exactly what to expect from psychotherapy** | 61.88 | 31.38 | .82 |
| **I do not know what I can safely disclose in therapy < > I know exactly what I can safely disclose in therapy** | 53.97 | 36.55 | .68 |
| **I do not know a lot about different treatment approaches < > I know a lot about different treatment approaches** | 53.26 | 32.09 | .75 |
