# Supplementary material for: Stigma processes, psychological distress, and attitudes toward seeking treatment among pedohebephilic people
Source: PLoS One. 2024 Oct 24;19(10):e0312382. doi: 10.1371/journal.pone.0312382 (PMC11500907; doi:10.1371/journal.pone.0312382)
Supplement: S5 Table — (DOCX) [file pone.0312382.s005.docx]

**S5 Table. Model parameters for model 1 (pre-registered model).**

|  | **Parameter estimate (Standard error)** |
| --- | --- |
|  | **Factor Loadings** |
| Attitudes Toward Seeking Professional Psychological Help Scale (latent) |  |
| Openness to Seeking Professional Help (latent) | 1.00+ |
| Value in Seeking Professional Help (latent) | 1.05(0.12)** |
| Preference to Cope on One’s Own (latent) | 0.39(0.09)** |
| Openness to Seeking Professional Help (latent) |  |
| Attitudes Toward Seeking Professional Psychological Help Scale item 1 | 1.00+ |
| Attitudes Toward Seeking Professional Psychological Help Scale item 5 | 1.28(0.11)** |
| Attitudes Toward Seeking Professional Psychological Help Scale item 6 | 1.39(0.12)** |
| Attitudes Toward Seeking Professional Psychological Help Scale item 7 | 0.79(0.10)** |
| Value in Seeking Professional Help (latent) |  |
| Attitudes Toward Seeking Professional Psychological Help Scale item 2 (reverse-coded) | 1.00+ |
| Attitudes Toward Seeking Professional Psychological Help Scale item 3 | 1.06(0.08)** |
| Attitudes Toward Seeking Professional Psychological Help Scale item 8 (reverse-coded) | 0.76(0.10)** |
| Preference to Cope on One’s Own (latent) |  |
| Attitudes Toward Seeking Professional Psychological Help Scale item 4 (reverse-coded) | 1.00+ |
| Attitudes Toward Seeking Professional Psychological Help Scale item 9 (reverse-coded) | 1.97(0.33)** |
| Attitudes Toward Seeking Professional Psychological Help Scale item 10 (reverse-coded) | 1.19(0.22)** |
| Anticipated Therapist Behavior Upon Disclosure (latent) |  |
| Reduced Collaboration/ Respect (latent) | 1.00+ |
| Adversarial Therapist Behavior (latent) | 0.98(0.06)** |
| Treatment Discontinuation (latent) | 0.95(0.06)** |
| Reduced Collaboration/ Respect (latent) |  |
| treat.resp | 1.00+ |
| pressure.int | 0.78(0.06)** |
| appreciate | 0.68(0.06)** |
| pay.attention | 0.87(0.05)** |
| Adversarial Therapist Behavior (latent) |  |
| report | 1.00+ |
| hurtful | 1.09(0.06)** |
| stop.trusting | 1.17(0.06)** |
| ridicule | 0.84(0.06)** |
| Treatment Discontinuation (latent) |  |
| cannot.work.longer | 1.00+ |
| continue.see | 0.79(0.04)** |
| reject | 1.07(0.05)** |
| Knowledge about Psychotherapy Scale (latent) |  |
| Knowledge about Psychotherapy Scale item 1 (re-scaled) | 1.00+ |
| Knowledge about Psychotherapy Scale item 2 (re-scaled) | 0.98(0.08)** |
| Knowledge about Psychotherapy Scale item 3 (re-scaled) | 1.14(0.07)** |
| Knowledge about Psychotherapy Scale item 4 (re-scaled) | 1.12(0.09)** |
| Knowledge about Psychotherapy Scale item 5 (re-scaled) | 1.07(0.08)** |
| Internalizing Symptoms Regarding Minor-Attraction |  |
| Internalizing Symptoms Regarding Minor-Attraction subscale item 1 | 1.00+ |
| Internalizing Symptoms Regarding Minor-Attraction subscale item 2 | 1.37(0.10)** |
| Internalizing Symptoms Regarding Minor-Attraction subscale item 3 (reverse-coded) | 0.95(0.10)** |
| Internalizing Symptoms Regarding Minor-Attraction subscale item 4 | 1.25(0.09)** |
| Internalizing Symptoms Regarding Minor-Attraction subscale item 5 | 0.81(0.11)** |
| Internalizing Symptoms Regarding Minor-Attraction subscale item 6 (reverse coded) | 1.17(0.09)** |
| Internalizing Symptoms Regarding Minor-Attraction subscale item 8.rec | 1.26(0.11)** |
| Fear of Rejection/Concealment subscale (latent) |  |
| Fear of Rejection/Concealment subscale item 1 | 1.00+ |
| Fear of Rejection/Concealment subscale item 2 | 1.11(0.09)** |
| Fear of Rejection/Concealment subscale item 3 | 0.85(0.07)** |
| Fear of Rejection/Concealment subscale item 7 | 0.79(0.08)** |
| Fear of Rejection/Concealment subscale item 9 | 0.98(0.10)** |
| Psychological Distress (latent) |  |
| Somatization (latent) | 1.00+ |
| Depression (latent) | 2.24(0.34)** |
| Anxiety (latent) | 2.26(0.34)** |
| Somatization (latent) |  |
| Brief Symptom Inventory-18 item 1 | 1.00+ |
| Brief Symptom Inventory-18 item 4 | 0.98(0.15)** |
| Brief Symptom Inventory-18 item 7 | 1.09(0.15)** |
| Brief Symptom Inventory-18 item 10 | 1.13(0.17)** |
| Brief Symptom Inventory-18 item 13 | 1.11(0.16)** |
| Brief Symptom Inventory-18 item 16 | 1.38(0.18)** |
| Depression (latent) |  |
| Brief Symptom Inventory-18 item 2 | 1.00+ |
| Brief Symptom Inventory-18 item 5 | 0.99(0.09)** |
| Brief Symptom Inventory-18 item 8 | 1.02(0.08)** |
| Brief Symptom Inventory-18 item 11 | 1.37(0.10)** |
| Brief Symptom Inventory-18 item 14 | 1.39(0.10)** |
| Brief Symptom Inventory-18 item 17 | 1.01(0.09)** |
| Anxiety (latent) |  |
| Brief Symptom Inventory-18 item 3 | 1.00+ |
| Brief Symptom Inventory-18 item 6 | 0.87(0.05)** |
| Brief Symptom Inventory-18 item 9 | 0.94(0.06)** |
| Brief Symptom Inventory-18 item 12 | 0.90(0.06)** |
| Brief Symptom Inventory-18 item 15 | 0.57(0.08)** |
| Brief Symptom Inventory-18 item 18 | 0.97(0.06)** |
| WHO-5 Wellbeing index (latent) |  |
| WHO-5 Wellbeing index item 1 (reverse-coded) | 1.00+ |
| WHO-5 Wellbeing index item 2 (reverse-coded) | 0.87(0.05)** |
| WHO-5 Wellbeing index item 3 (reverse-coded) | 0.85(0.05)** |
| WHO-5 Wellbeing index item 4 (reverse-coded) | 0.90(0.06)** |
| WHO-5 Wellbeing index item 5 (reverse-coded) | 0.91(0.05)** |
|  | **Regression Slopes** |
| Attitudes Toward Seeking Professional Psychological Help Scale (latent) |  |
| Fear of Rejection/Concealment subscale (latent) | -0.06(0.04) |
| Internalizing Symptoms Regarding Minor-Attraction | 0.20(0.05)** |
| Anticipated Therapist Behavior Upon Disclosure (latent) | -0.12(0.03)** |
| Knowledge about Psychotherapy Scale (latent) | 0.07(0.02)** |
| Psychological Distress (latent) | 0.49(0.22)* |
| WHO-5 Wellbeing index (latent) | 0.18(0.07)** |
| Anticipated Therapist Behavior Upon Disclosure (latent) |  |
| Knowledge about Psychotherapy Scale (latent) | -0.18(0.05)** |
| Psychological Distress (latent) |  |
| Internalizing Symptoms Regarding Minor-Attraction | 0.19(0.04)** |
| Fear of Rejection/Concealment subscale (latent) | -0.06(0.02)* |
| WHO-5 Wellbeing index (latent) |  |
| Internalizing Symptoms Regarding Minor-Attraction | -0.50(0.07)** |
| Fear of Rejection/Concealment subscale (latent) | 0.14(0.07)* |
|  | **Residual Variances** |
| Attitudes Toward Seeking Professional Psychological Help Scale item 1 | 0.75(0.06)** |
| Attitudes Toward Seeking Professional Psychological Help Scale item 5 | 0.49(0.06)** |
| Attitudes Toward Seeking Professional Psychological Help Scale item 6 | 0.50(0.06)** |
| Attitudes Toward Seeking Professional Psychological Help Scale item 7 | 0.54(0.05)** |
| Attitudes Toward Seeking Professional Psychological Help Scale item 2 (reverse-coded) | 0.35(0.06)** |
| Attitudes Toward Seeking Professional Psychological Help Scale item 3 | 0.34(0.05)** |
| Attitudes Toward Seeking Professional Psychological Help Scale item 8 (reverse-coded) | 0.85(0.08)** |
| Attitudes Toward Seeking Professional Psychological Help Scale item 4 (reverse-coded) | 0.87(0.07)** |
| Attitudes Toward Seeking Professional Psychological Help Scale item 9 (reverse-coded) | 0.17(0.08)* |
| Attitudes Toward Seeking Professional Psychological Help Scale item 10 (reverse-coded) | 0.44(0.05)** |
| treat.resp | 1.10(0.15)** |
| pressure.int | 1.82(0.22)** |
| appreciate | 1.67(0.15)** |
| pay.attention | 1.09(0.18)** |
| report | 1.69(0.20)** |
| hurtful | 1.42(0.16)** |
| stop.trusting | 1.11(0.14)** |
| ridicule | 1.59(0.16)** |
| cannot.work.longer | 0.97(0.14)** |
| continue.see | 1.10(0.16)** |
| reject | 0.79(0.14)** |
| Knowledge about Psychotherapy Scale item 1 (re-scaled) | 2.70(0.34)** |
| Knowledge about Psychotherapy Scale item 2 (re-scaled) | 5.68(0.68)** |
| Knowledge about Psychotherapy Scale item 3 (re-scaled) | 3.34(0.48)** |
| Knowledge about Psychotherapy Scale item 4 (re-scaled) | 7.12(0.68)** |
| Knowledge about Psychotherapy Scale item 5 (re-scaled) | 4.56(0.50)** |
| Internalizing Symptoms Regarding Minor-Attraction subscale item 1 | 2.39(0.23)** |
| Internalizing Symptoms Regarding Minor-Attraction subscale item 2 | 1.68(0.19)** |
| Internalizing Symptoms Regarding Minor-Attraction subscale item 3 (reverse-coded) | 1.90(0.23)** |
| Internalizing Symptoms Regarding Minor-Attraction subscale item 4 | 1.56(0.18)** |
| Internalizing Symptoms Regarding Minor-Attraction subscale item 5 | 3.60(0.27)** |
| Internalizing Symptoms Regarding Minor-Attraction subscale item 6 (reverse coded) | 1.40(0.17)** |
| Internalizing Symptoms Regarding Minor-Attraction subscale item 8.rec | 1.70(0.20)** |
| Fear of Rejection/Concealment subscale item 1 | 1.57(0.25)** |
| Fear of Rejection/Concealment subscale item 2 | 0.99(0.15)** |
| Fear of Rejection/Concealment subscale item 3 | 1.11(0.20)** |
| Fear of Rejection/Concealment subscale item 7 | 0.62(0.10)** |
| Fear of Rejection/Concealment subscale item 9 | 1.41(0.23)** |
| Brief Symptom Inventory-18 item 1 | 0.50(0.06)** |
| Brief Symptom Inventory-18 item 4 | 0.50(0.06)** |
| Brief Symptom Inventory-18 item 7 | 0.60(0.08)** |
| Brief Symptom Inventory-18 item 10 | 0.44(0.06)** |
| Brief Symptom Inventory-18 item 13 | 0.49(0.09)** |
| Brief Symptom Inventory-18 item 16 | 0.55(0.08)** |
| Brief Symptom Inventory-18 item 2 | 0.78(0.07)** |
| Brief Symptom Inventory-18 item 5 | 1.05(0.08)** |
| Brief Symptom Inventory-18 item 8 | 0.72(0.08)** |
| Brief Symptom Inventory-18 item 11 | 0.61(0.08)** |
| Brief Symptom Inventory-18 item 14 | 0.64(0.07)** |
| Brief Symptom Inventory-18 item 17 | 0.66(0.08)** |
| Brief Symptom Inventory-18 item 3 | 0.54(0.06)** |
| Brief Symptom Inventory-18 item 6 | 0.67(0.08)** |
| Brief Symptom Inventory-18 item 9 | 0.56(0.07)** |
| Brief Symptom Inventory-18 item 12 | 0.49(0.06)** |
| Brief Symptom Inventory-18 item 15 | 0.97(0.11)** |
| Brief Symptom Inventory-18 item 18 | 0.52(0.07)** |
| WHO-5 Wellbeing index item 1 (reverse-coded) | 0.42(0.06)** |
| WHO-5 Wellbeing index item 2 (reverse-coded) | 0.67(0.08)** |
| WHO-5 Wellbeing index item 3 (reverse-coded) | 0.70(0.08)** |
| WHO-5 Wellbeing index item 4 (reverse-coded) | 1.01(0.10)** |
| WHO-5 Wellbeing index item 5 (reverse-coded) | 0.87(0.09)** |
|  | **Latent Variances** |
| Attitudes Toward Seeking Professional Psychological Help Scale (latent) | 0.26(0.05)** |
| Openness to Seeking Professional Help (latent) | 0.01(0.03) |
| Value in Seeking Professional Help (latent) | 0.17(0.06)** |
| Preference to Cope on One’s Own (latent) | 0.11(0.04)** |
| Anticipated Therapist Behavior Upon Disclosure (latent) | 2.26(0.24)** |
| Reduced Collaboration/ Respect (latent) | 0.58(0.14)** |
| Adversarial Therapist Behavior (latent) | 0.08(0.10) |
| Treatment Discontinuation (latent) | 0.25(0.09)** |
| Knowledge about Psychotherapy Scale (latent) | 4.99(0.68)** |
| Internalizing Symptoms Regarding Minor-Attraction | 1.79(0.27)** |
| Fear of Rejection/Concealment subscale (latent) | 1.62(0.26)** |
| Psychological Distress (latent) | 0.09(0.03)** |
| Somatization (latent) | 0.17(0.04)** |
| Depression (latent) | 0.04(0.03) |
| Anxiety (latent) | 0.32(0.06)** |
| WHO-5 Wellbeing index (latent) | 0.99(0.11)** |
|  | **Latent Covariances** |
| Internalizing Symptoms Regarding Minor-Attraction w/Fear of Rejection/Concealment subscale (latent) | 0.87(0.15)** |
| Anticipated Therapist Behavior Upon Disclosure (latent) w/Fear of Rejection/Concealment subscale (latent) | 0.29(0.12)* |
| Anticipated Therapist Behavior Upon Disclosure (latent) w/Internalizing Symptoms Regarding Minor-Attraction | 0.36(0.13)** |
| Psychological Distress (latent) w/WHO-5 Wellbeing index (latent) | -0.21(0.04)** |
| Knowledge about Psychotherapy Scale (latent) w/Internalizing Symptoms Regarding Minor-Attraction | -0.15(0.21) |
| Knowledge about Psychotherapy Scale (latent) w/Fear of Rejection/Concealment subscale (latent) | -0.67(0.20)** |

* *p* < .05, ** *p* > .01
