# Supplementary material for: Stigma processes, psychological distress, and attitudes toward seeking treatment among pedohebephilic people
Source: PLoS One. 2024 Oct 24;19(10):e0312382. doi: 10.1371/journal.pone.0312382 (PMC11500907; doi:10.1371/journal.pone.0312382)
Supplement: S1 File — (DOCX) [file pone.0312382.s007.docx]

File S1. Deviations from the preregistration

**Date: June 2024**

**General information:** This project was preregistered as research question 2 (of 3) on January 4, 2021. The preregistration is available on OSF: <https://osf.io/kpza6/>

This document will follow the structure of the preregistration, commenting on all deviations that have been conducted and assessing them with respect to their putative effects on the severity of the test and the validity of the inference (Lakens, 2024).

VARIABLES

1. What are your independent /grouping/ predictor variables?

All variables stated in the preregistration have been used in our analyses. No predictor variable was dropped from the correlation matrix in Table 1 or from Model 1 (i.e., the first SEM model). Hence, all pre-registered hypotheses were tested, both with binary correlations and within a SEM.

However, we modified some of the new scales or the scales that have not been pre-tested in more than one prior study in situations where auxiliary assumption about the factor structure of included variables were falsified. Moreover, we have changed our terminology when referring to the variables in our research for many scales. These deviations will be listed and evaluated below:

| **Type of deviation/ Variable** | **Deviation(s)** | **Reason** | **Risk of bias** |
| --- | --- | --- | --- |
| **Terminological change** |  |  | none |
| Subscale Fear of Detection/Rejection | General anticipated stigma (note that the name of the subscale and the scale have also been changed to Fear of Rejection/ Concealment and Proximal Stigmas Scale for Minor Attracted People by the authors of the scale in the time between the preregistration and the submission of the article) | to align more closely with the theoretical framework | none |
| Internalizing Symptoms regarding minor-attraction | Internalized Stigma | to align more closely with the theoretical framework | none |
| Expected Therapist Malpractice Upon Disclosure | Anticipated therapist behavior upon disclosure | to align more closely with the theoretical framework | none |
| Subscale Internalizing Symptoms | Internalized stigma | to align more closely with the theoretical framework | none |
| Knowledge about mental health services | Knowledge about psychotherapy | To align more closely with the item formulations | none |
| **Modified SEM (Model 2)** |  |  |  |
| Psychological wellbeing (WHO-5 Well-being index)^[[1]](#footnote-1)^ | Deleted in Model 2 | Strong conceptual overlap between the WHO-5 and the BSI-18, which led to problems with interpretability, as described in the article. | Low. The direction and significance of all remaining regression paths are the same in Model 1 (which includes all variables) and Model 2. |

1. What are your dependent / outcome variables?

All outcome variables stated in the preregistration have been used in our analyses. No outcome variable was dropped from the correlation matrix or from Model 1 (i.e., the first SEM). Psychological wellbeing was deleted in Model 2 (see Section 1 for a more detailed description).

3. List any exploratory variables. These are variables that you included in your study, but are not central to your main predictions

We did not include any of the variables listed as potential exploratory variables.

HYPOTHESES

What are your primary study hypotheses / research questions? Do you have any exploratory hypotheses / research questions?

As preregistered, we have conducted confirmatory factor analyses for all scales that were self-developed for the current project or that have not been pre-tested in more than one prior study, assuming one-factor solutions for each (1.1). We tested all preregistered bivariate correlations specified in the hypothesis section (1.2). Furthermore, we tested all multivariate hypotheses (including indirect effects) using SEM (1.3).

In Model 2, due to the deletion of psychological wellbeing, the indirect effects were only tested for the variables that remained in the model.

SAMPLING

| **Type of deviation** | **Deviation(s)** | **Reason** | **Risk of bias** |
| --- | --- | --- | --- |
| Stopping rule | It was preregistered to terminate data collection after 5 months, in case that the recruitment could not be reached before that. Data collection was started on January 7^th^ and terminated exactly one month too early (on May 6th) | By mistake. Data collection was stopped before the beginning of the fifth month, not after 5 months have passed. | None, as the decision occurred by miscalculating the months, not based on the data or results. |

RESEARCH DESIGN

The preregistration describes the project as a correlational study with quantitative and qualitative components (the latter of which do not apply to research question 2). No deviations from the preregistration.

DATA ANALYSIS PART

All exclusion criteria regarding sexual interests, age, honesty and seriousness checks, and completion time were followed. In line with preregistrations, no outliers were removed. We employed the tests we preregistered for this Research Question 2: Confirmatory Factor Analyses, Correlations, Structural equation models. The initial modification strategy for the SEM analyses specified that items can be removed (if factor loading on latent trait < .40), residual correlations between items specified (if indicated by the model fit and modification indices following standard criteria), and scales removed/collapsed if correlations with other scales are > .80. In hindsight, it is unfortunate that limits for modification indices were not pre-specified, as this left a high degree of researcher freedom. Changes were made with regards to the use of FIML and the SEM modification strategy, as described below:

| **Type of deviation** | **Deviation(s)** | **Reason** | **Risk of bias** |
| --- | --- | --- | --- |
| Dealing with missing values | Use of FIML to deal with missing values in the full SEM | FIML is only available for traditional ML models, not for ML models with a Satorra-Bentler-corrected test statistic. After assessing the data, we realized that some items deviated from the assumption of normality, which can affect the validity of the SEM analyses. As missingness was negligible, we decided to use the Satorra-Bentler-corrected test statistic | Low |
| Modification criterion | CFI > .90 for all models,  RMSEA < .08 (CFI will be given more weight in models with small df) | We did not preregister thresholds for acceptable model fit – this was an oversight. | Medium |
| Modification strategy | Use of UVA for scales with bad model fit | Unforeseen problems with the anticipated negative therapist behavior upon disclosure scale | **Medium for hypotheses about links between factors**, as it was not preregistered how to proceed in case a unidimensional model cannot be attained. However, the higher order factor model including all items was aligned with our original idea of Anticipated Therapist Behavior upon disclosure as a unidimensional construct.  **High for the factor structure of the instrument itself**: It was expected that the scale would be unidimensional. Yet, fit indices indicated a better fit for a three-level model. A new study is needed to determine whether the three-factor structure will replicate. |

**References**:

Lakens, D. (2024). When and how to deviate from a preregistration. *Collabra: Psychology*, *10*(1).

1. Note that WHO-5 functions both as a predictor and an outcome variable in the SEM model. To avoid redundancy, it is only mentioned here and not in 1.2 [↑](#footnote-ref-1)
