# Supplementary material for: Stigma processes, psychological distress, and attitudes toward seeking treatment among pedohebephilic people
Source: PLoS One. 2024 Oct 24;19(10):e0312382. doi: 10.1371/journal.pone.0312382 (PMC11500907; doi:10.1371/journal.pone.0312382)
Supplement: Attachment — Submitted filename: PLOSOne Letter to the reviewers.docx [file pone.0312382.s009.docx]

Dear Dr. Schalinski,

Thank you for inviting us to submit a revised version of the manuscript that addresses the points raised during the review process. We want to express our sincere gratitude to the reviewers for providing such clear and constructive feedback. Below, please find a detailed response to each of the points raised by the editor and the three reviewers. We have provided both a version of our manuscript with track changes and a version without track changes.

**Sincerely,**

**Sara Jahnke,**

**Ian McPhail,**

**Jan Antfolk**

**####Editor:**

**>>I have received three excellent and thorough reviews for your manuscript, two suggesting major revisions and 1 suggesting minor revisions. Reviewer 1 emphasized the need for a more detailed discussion to enhance its depth. Reviewer 2 suggested more comprehensive methodological revisions to ensure thoroughness. Additionally, Reviewer 3 recommended further clarification of the analytic protocol, pinpointing areas for improvement. With these precise and constructive reviews, you can refine your manuscript effectively, ensuring clarity and rigor in your research before acceptance.**

**Response:** Thank you for considering our manuscript and finding three reviewers with expertise in our field of work. We agree with you that all wrote excellent reviews and were impressed by the quality of their feedback not just on our manuscript, but also the affiliated documents. We have now addressed each of the reviewers’ points below.

**####Journal Requirements:**

**>>1. When submitting your revision, we need you to address these additional requirements.**

**>>Please ensure that your manuscript meets PLOS ONE's style requirements, including those for file naming. The PLOS ONE style templates can be found at** 
[**https://journals.plos.org/plosone/s/file?id=wjVg/PLOSOne_formatting_sample_main_body.pdf**](https://journals.plos.org/plosone/s/file?id=wjVg/PLOSOne_formatting_sample_main_body.pdf) **and** 
[**https://journals.plos.org/plosone/s/file?id=ba62/PLOSOne_formatting_sample_title_authors_affiliations.pdf**](https://journals.plos.org/plosone/s/file?id=ba62/PLOSOne_formatting_sample_title_authors_affiliations.pdf)

**Response:** We have made the necessary changes based on the two style templates that were provided and included new file names for the Supplementary Material.

>>2. We note that the grant information you provided in the ‘Funding Information’ and ‘Financial Disclosure’ sections do not match.

>>When you resubmit, please ensure that you provide the correct grant numbers for the awards you received for your study in the ‘Funding Information’ section.

**Response:** Done. 333761 is the correct application/decision number for the grant. I did not receive any other number in relation to this project from the Academy of Finland.

>>3. Thank you for stating the following financial disclosure: 
This study was funded by a personal grant (application number 333761) to the first author from the Academy of Finland.

>>Please state what role the funders took in the study.  If the funders had no role, please state: "The funders had no role in study design, data collection and analysis, decision to publish, or preparation of the manuscript." 
If this statement is not correct you must amend it as needed. 
Please include this amended Role of Funder statement in your cover letter; we will change the online submission form on your behalf.

**Response:** Done. We added that "The funders had no role in study design, data collection and analysis, decision to publish, or preparation of the manuscript."

>>4. In the online submission form you indicate that your data is not available for proprietary reasons and have provided a contact point for accessing this data. Please note that your current contact point is a co-author on this manuscript. According to our Data Policy, the contact point must not be an author on the manuscript and must be an institutional contact, ideally not an individual. Please revise your data statement to a non-author institutional point of contact, such as a data access or ethics committee, and send this to us via return email. Please also include contact information for the third party organization, and please include the full citation of where the data can be found.

**Response: We have archived the dataset in** Surveybanken where it will be available to researchers upon request. <https://doi.org/10.18712/NSD-NSD3178-V2>

Note that this is the complete dataset, which also includes variables that have not been used in the present research.

>>5. Thank you for uploading your study's underlying data set. Unfortunately, the repository you have noted in your Data Availability statement does not qualify as an acceptable data repository according to PLOS's standards.

>>At this time, please upload the minimal data set necessary to replicate your study's findings to a stable, public repository (such as figshare or Dryad) and provide us with the relevant URLs, DOIs, or accession numbers that may be used to access these data. For a list of recommended repositories and additional information on PLOS standards for data deposition, please see <https://journals.plos.org/plosone/s/recommended-repositories>.

**Response:** We have added a doi to the Data Availability Statement, which now reads as follows: “The raw data are available for researchers upon request via the database surveybanken, provided by the Norwegian Agency for Shared Services in Education and Research (Sikt).This is in line with information provided to participants during the informed consent procedures, as publication of the dataset is likely to deter pedohebephilic people from participating in research studies due to fears of being identified. ”

Citation: Jahnke, Sara. (2024). Better therapy for people who are sexually attracted to children: A survey on labels, treatment motivation, and treatment experiences [Data set]. Sikt - Norwegian Agency for Shared Services in Education and Research. <https://doi.org/10.18712/NSD-NSD3178-V2>

>>6. We note that you have referenced McPhail IV, Stephens S. which has currently not yet been accepted for publication. Please remove this from your References and amend this to state in the body of your manuscript: (McPhail IV, Stephens S. [Submitted]) as detailed online in our guide for authors
[http://journals.plos.org/plosone/s/submission-guidelines#loc-reference-style](http://journals.plos.org/plosone/s/submission-guidelines#loc-reference-style )

**Response: As the manuscript you are referring to has now been published, we have amended the section accordingly.**

>>7. We note that you have referenced Chronos A, Jahnke S, Blagden N. which has currently not yet been accepted for publication. Please remove this from your References and amend this to state in the body of your manuscript: (Chronos A, Jahnke S, Blagden N. [Submitted]) as detailed online in our guide for authors
[http://journals.plos.org/plosone/s/submission-guidelines#loc-reference-style](http://journals.plos.org/plosone/s/submission-guidelines#loc-reference-style )

**Response: The manuscript has now been accepted for publication, and the citation has been amended accordingly.**

**####Reviewer #1:**

**>>**I enjoyed your paper.

**Response: Thank you! We are glad to hear that.**

>>The main problem I found is that there are two important, recent studies on this topic that are ignored in the article: Roche and Stephens, 2022 (“Clinician stigma and willingness to treat those with sexual interest in children”), and Schmidt and Niehaus, 2022 (“Outpatient Therapists’ Perspectives on Working with Persons Who Are Sexually Interested in Minors”). Once we consider findings from these two studies, your claim that “extreme stigmatization of pedophilic clients appears to be rare among therapists” should be much more nuanced, and your discussion (starting at line 477) about the accuracy of pedophebephilic individuals’ beliefs and anticipations of negative attitudes from therapists should be different.

>>Roche and Stephens, 2022 found that 48% of therapists from their sample did not want to work with pedophebephilic individuals who had a past offense and wanted help managing their attraction. Schmidt and Niehaus, 2022 found that 20% of their Swiss therapists believed that pedophebephilic individuals were destined to sexually abuse a child; 40% said that ALL pedophebephilic individuals should be mandated to receive psychotherapy, and 57% agreed with a statement saying that people who are attracted to children are “sick”.

**Response: The disagreement seems to hinge on our diverging interpretation of the word “extreme stigmatization”**. I am aware of the numbers reported in Schmidt & Niehaus and Roche & Stephens. In fact, the sentence you quoted ends in a citation referring Niehaus et al. (2020), which is a report based on the same dataset and with the same information that is covered in Schmidt & Niehaus, 2022 (note I have changed the reference to the peer-reviewed article in the new version of the manuscript). My reading of the report/paper was more optimistic, given that agreement with chemical castration, preventive detention, and sex offender registries was uncommon and much less frequent in the professional sample compared to the general public. As a compromise, we changed the section as follows:

“By all accounts, extreme stigmatization of pedophilic clients appears to be less common among therapists than the general public (62). Nevertheless, 40% of Swiss therapists agreed that all pedohebephilic individuals should receive mandated treatment and one in five believed that pedohebephilic individuals are destined to commit child sexual abuse (62). There is also evidence that stigmatizing attitudes can inform decision-making by clinicians, including making mandated reports in situations with pedohebephilic clients that do not warrant a report (63) or their willingness to offer services to this client population (64).”

We did not include the treatment willingness rates reported in Roche & Stephens. First of all, the study is based on a small sample (N = 101) where only a quarter (n = 27) of the participants have received the vignette about the pedohebephilic client looking for help to manage sexual interests. Interestingly, we also do not think that this paper contradicts our claims about extreme stigmatization being rare in clinician samples. We would not have expected 52% to be willing to treat a client who has offended and is seeking help to learn to manage their sexual interests. With all vignettes combined, almost three-quarters were willing to offer treatment, despite the fact that many are likely to lack fundamental expertise in the area. Given that this was a generalist sample, this rate strikes us as surprisingly high. In line with Roche & Stephens, we agree that “we do not want to imply that clinicians should work outside their area of competency” and also believe that “it is a problem that many clinicians are not receiving education about clients with sexuality-based concerns” (p. 9). The 48% that declined to treat may partially or even entirely consist of therapists who do not want to treat clients outside of their area of competence, which constitutes a requirement of ethical practice. Nevertheless, we are now referencing the study in a related context, to back up the claim that “there is also evidence that stigmatizing attitudes can inform decision-making by clinicians, including […] their willingness to offer services to this client population (64).”

>>Some of these findings need to be mentioned, and your interpretation in the discussion section needs to be modified accordingly. Similarly, your recommendation to develop education campaigns aiming to “decrease fears of negative therapist behavior” among pedophebephilic individuals seems premature in light of these other findings. Such education campaigns are first and foremost needed to change therapists’ views about pedophebephilic individuals.

**Response:** We understand the reviewers’ concern. In fact, we agree that being afraid of negative therapist behavior is not at all unreasonable, as evidenced by this recent publication that some of us (co-)authored (Jahnke et al., 2023). Our actual stance on this issue is more nuanced, as indicated by the second part of the sentence, which states that such education needs to be combined with education about “strategies to identify therapists who are less likely to react negatively.” To avoid misunderstandings, we have changed the section as follows:

“Hence, we would suggest an intervention that combines education about pedohebephilia with interventions that challenge the assumptions that therapists who would be willing and capable to provide high quality care do not exist. Previous research has shown that intergroup contact interventions, even when delivered indirectly via video, can improve outgroup attitudes and behaviors (e.g., 68). An intervention to increase trust in therapists could include videos or stories involving a warm, non-judgmental therapist. As a caveat, such an intervention would also need to acknowledge that some therapists do react unethically upon learning that their client is sexually attracted to children. Therefore, it is of utmost importance that the intervention would also provide strategies to help potential clients identify therapists who possess the necessary skills and attitudes to work with this client group.”

Jahnke, S., Blagden, N., Mcphail, I. V., & Antfolk, J. (2023). Secret-keeping in therapy by clients who are sexually attracted to children. Psychotherapy Research, 1-16.

>>I have other more minor points.

>>On lines 97 and 98, you write: “Studies on the link between anticipating rejection and seeking treatment in pedophebephilic individuals have produced mixed results (8).” The reference to (8) here seems to be wrong. It links to Clement et al. (2015), which does not discuss pedophebephilic individuals.

**Response: Thank** you for catching this oversight! We have changed the text as follows: “Studies on the link between anticipating rejection and seeking treatment for a mental health problem have produced mixed results (8).”

>>In relation to the previous point on anticipating rejection: I am surprised that there is no mention of the often-cited B4U-ACT 2011 survey results here. In that survey, the top reason given for MAPs not seeking professional help is: "Fear of negative reaction by the professional (78%)".

**Response: This refers to a survey conducted and published by the patient advocacy group B4U-ACT on their webpage. The first author of this manuscript has cited the B4U-ACT 2011 survey in a previous paper, at a time when peer-reviewed research on barriers to treatment was not available in the academic literature. This is not the case anymore. In the manuscript, we write that “Similar processes may be at work among pedohebephilic individuals, as anticipation of being rejected or being unnecessarily reported to the authorities by clinicians are frequently reported barriers to seeking treatment (11,26)” citing**

- Levenson JS, Grady MD. Preventing sexual abuse: Perspectives of minor-attracted persons about seeking help. Sex Abuse. 2019;1079063218797713.
- Houtepen JABM, Sijtsema JJ, Bogaerts S. Being sexually attracted to minors: Sexual development, coping with forbidden feelings, and relieving sexual arousal in self-identified pedophiles. Journal of Sex and Marital Therapy. 2015 Jun 22;48–69.

Hence, recent peer-reviewed literature on the topic of barriers is available and included in the article.

>>Finally, in various places, the expression “sexual minorities” is used, and its use seems to include LGB populations but exclude pedophebephilic individuals (example of exclusion on line 475: "(e.g.,sexual minority or pedohebephilia)”. It’s not clear at all why pedophebephilic individuals wouldn’t count as a “sexual minority", and you
never define the expression. To avoid confusion, you should either 1) drop the expression “sexual minority” and stick to LGB or 2) drop the opposite between pedophebephilic individuals and sexual minorities.

**Response: Such language in reference to people with a sexual interest in children has been considered highly inflammatory by a reviewer of a previous article by the first author of this manuscript. At the same time, we do agree that it is strange to exclude pedohebephilic people from the sexual minority label. Therefore, we opted for 1) and dropped the expression “sexual minority” from the text.**

**####Reviewer #2:**

**>>**The current article investigates a number of important scales with respect to the motivation of pedohebephilic patients to seek treatment. This is a very important topic, and it is of great importance that the authors work on scales that are crucial for other authors to research this topic. The authors on top of that present an interesting mediation result that may explain differing previous results in the literature. There are a number of issues with the methodology used or the presentation of the methodology, at some points I admit I’m not sure which of the two. To make sure that the important results are not hidden behind these problems, I think the manuscript would benefit from some clarification here before publication. The only major change I would suggest is to remove the WLSMV estimator from the article since it is theoretically less founded, created some practical problems with this data set which forced to remove two subscales, and other detail reasons I outline below.

**Response: Thank you! We have removed the WLSMV estimator, for the practical reasons that you mentioned. This is justified in the paper as follows:**

**“Treating ordinal variables as continuous may lead to imprecise parameter estimates and standard errors (50). As the scale assessing attitudes toward seeking treatment only includes four response alternatives (50,51), we considered using weighted least squares means and variance adjusted (WLSMV) estimator, which is more adequate for ordinal data (52), as a sensitivity test. However, WLSMV may perform worse than ML in small samples and nonnormal latent distributions (53). Furthermore, the use of WLSMV created identification problems in the current data, which led to the removal of two subscales from the main outcome (attitudes toward seeking treatment). Therefore, the present study will only perform ML.”**

>>Major Issues:

>>line 217-220: The scale that participants were able to answer from is missing. It seems from the means in Table S7 that items were on a scale from 1 to 100, but that doesn’t seem to match a total score from 1 to 10. Computing ordinal scores that correspond to ranges suggest a five-point Likert scale. You may want to clarify.

**Response: Thank you for pointing this out to us. We understand the confusion. The original scale (as presented to participants) went from 1 to 100, but it was not possible to include these values in the ML model. Due to wanting homogeneous ranges as a prerequisite for modeling, we transformed the scale to go from 1 to 10 (in 0.1 steps). This should have been explained more clearly. We hope that the section is clearer now:**

**“Participants responded to items on a 100-point visual analog scale. Scalar anchors were statements reflecting a low and high level of knowledge of a specific psychotherapy topic/process (e.g., I do not know about my rights as a patient and I know a lot about my rights as a patient; see Table S4 for full item list). The scores were re-scaled from an initial range between 1 and 101 to a range between 0 and 10 (by dividing all values by 10 and subtracting 0.1) to create a scale that is more in line with the ranges of the other scales in the subsequent SEM model.”**

>>line 297: Which missingness treatment strategy did you use instead of FIML for the remaining 2.8% missingness? It seems from the reduction of sample size that you used listwise deletion; note that this is introducing a bias (although admittedly probably small for only eight participants). In doubt, I guess FIML on uncorrected data ML is the better choice; you may want to simulate to be sure (or just do both and compare results, if the outcome is more or less the same anyway, who cares).

**Response: FIML** (or other missingness treatment strategies, for that matter) is/are not available for ML estimation with a Satorra-Bentler-corrected test statistic, unfortunately. From the lavaan documentation: “MLM: Maximum likelihood parameter estimates with conventional robust standard errors and a Satorra-Bentler scaled test statistic that are robust to non-normality. For complete data only.” As we were more worried about non-normality than about the <3% missings, we opted for this estimator as opposed to the normal ML procedure, which would have included FIML. This is stated in the manuscript as follows: “We could not conduct the pre-registered FIML procedure, as it is not available for ML estimation. The pre-registered (SEM) model (Model 1) was conducted with a sample size of 275 out of 283, indicating a rate of missing values of 2.8%. As this rate was considered negligible, no further missingness analyses were conducted.”

>>line 316: A fit of CFI = .97 for a factor model with five items is fully acceptable, the RMSEA is less useful for such a model. Adding it is reducing the dfs from 5 to 4, there’s not much space left to perfect fit. it increases overfitting, and it makes the interpretation difficult (since the factor scores can no longer be computed as a weighted sum of the item scores). I’d suggest to just go with the original model.

**Response: We included the following information in the Method section, “In models with small degrees of freedom, greater emphasis was placed on CFI than RMSEA (57).” We do no longer include a covariance term for the WHO-5 model. Note that the CFIs for all established scales have been removed from the manuscript.**

>>factor scale improvements: You seemed to choose very different strategies for improving the factor models, removing items, adding residual covariances, or changing the number of factors. All methods are absolutely fine, but you run in the danger of overfitting when applying many of those strategies, a little more so if you don’t give a fixed strategy in which order you try them and when already reacting on fairly good fit values just if they are a little bit over (although that is often not acknowledged) arbitrary cut-off values. My suggestion would be to fix a strategy, here would be the one I’d suggest: For all reasonable number of factors, delete all items with a standardized loading below a given threshold (or whenever CFI is improving, that is fair too), and then pick the number of factors that are most reasonable from a substantive point of view and within small range of the best CFI. A purist might say it’s still overfitting (and that is probably true), but it would drastically reduce that effect.

**Response: Thank you for this suggestion, which we adopted. The section in the paper now reads as follows:**

**“**Stage 1. Assessing the factor structure of all new scales

Initially, we verified that the newly developed or less established scales functioned adequately on their own, based on RMSEA and CFI criteria. Scales were eliminated or collapsed with other scales if correlations with other scales were above .80 and items with factor loadings below 0.40 were eliminated (58). Furthermore, we considered specifying residual correlations between items (if indicated by the model fit and modification indices following standard criteria). In one case, a new scale continued to deviate substantially from the threshold of CFI = .90., which prompted us to assess violations of the assumption of local independence. To detect locally dependent (i.e., redundant) items, we employed unique variable analysis, which detects local dependency in latent variable models without a priori knowledge about the internal structure (59). Weighted topological overlap-values above .20 for a pair of variables indicate that the assumption of local independence is violated. In these cases, we deleted one of the two variables, giving preference to items that were more general, had higher variance, and better covered the construct (59).

Stage 2. Testing associations

After confirming adequate fit for the measurement models of the new scales, bivariate correlations were assessed based on the latent variables that emerged from conducting separate CFAs for all included scales. SEM model were used to test all variables as part of a larger, multifactorial model, with mediation effects. If the model fit of the preregistered SEM model including all variables was below the CFI or RMSEA threshold, we picked those factors that are most reasonable from a theoretical point of view and within small range of the best CFI and RMSEA.”

>>line 321: You later report the three factors of the BSI as (I think) Somatization, Depression, and Anxiety; if I got that correct, I suggest to introduce the names here.

**Response: We have removed all CFIs for established scales, in line with our pre-registration. The three subscales are introduced in the Method section as follows, “The BSI-18 has three subscales, Depression, Anxiety, and Somatization, and a second-order factor (Global Severity Index) to determine general psychological distress (42).”**

>>Table 1 Footnote a: Why did you use average scores for the means suddenly? That seems surprising, considering that your factor weights are sometimes a good deal away from uniform distribution. Also (this very minor) a combined score from manifests is usually no longer called a manifest variable, it’s a computation of a latent.

**Response: We are now referring to the combined score as “average composite scores”. The reason to use average scores for the means is to give readers an idea about the central tendency of that scale in our sample, so that they would know whether participants tended to agree or disagree.**

>>Table 1 correlations: Why did you use the latent scores to compute the correlations? Wouldn’t it make more sense to fix the factor loadings and use the latent covariances in an SEM that includes all factors? Note that factor scores are good for means, but not so much for everything to do with variances. However, in case there is a strong reason for that, better than nothing certainly.

**Response: We included bivariate correlations because this enables us to identify subtle effects that might otherwise be obscured within a full SEM. For instance, comparing SEM paths and bivariate links helped us identify the suppression effect that led to the exclusion of the variable “psychological well-being”. Furthermore, our pre-registration includes bivariate analyses, which we are now describing more clearly,**

“The following binary hypotheses were pre-registered: We assumed zero-order associations between positive attitudes toward seeking treatment and 1) knowledge about psychotherapy, 2) general anticipated stigma, 3) internalized stigma, 4) anticipated negative therapist behavior upon disclosure, 5) psychological distress, and 6) psychological wellbeing. For anticipated negative therapist behavior, we specified that this link was expected to be negative. Additionally, we assumed that psychological wellbeing and psychological distress are linked to 7) general anticipated stigma (positive correlation for psychological wellbeing and negative correlation for psychological distress), and 8) **internalized stigma (positive correlation for psychological wellbeing and negative correlation for psychological distress). Furthermore, we pre-registered zero-order correlations between anticipated therapist maltreatment upon disclosure and 9) Knowledge about psychotherapy (negative association).**

**[…]**

**Stage 2. Testing associations.**

**Bivariate associations**

**The correlation matrix, as well as means and standard deviations of the average composite scores of all scales can be found in Table 1. Out of 11 pre-registered hypothesized associations, nine were statistically confirmed in our dataset. In line with our expectations, we detected zero-order associations between attitudes toward seeking treatment on the one hand and knowledge about psychotherapy, anticipated negative therapist behavior upon disclosure, internalized stigma, and psychological distress on the other hand. Against our expectations, no statistical associations were found between general anticipated stigma or psychological wellbeing on the one hand and attitudes toward seeking treatment on the other hand. For psychological distress, we corroborated the expected link to higher internalized stigma, but not to general anticipated stigma. Furthermore, we detected a link between psychological wellbeing and lower general anticipated stigma, as well as lower internalized stigma. The hypothesized negative link between anticipated therapist maltreatment upon disclosure Knowledge about psychotherapy was also confirmed.”**

>>Finally, you indicate the table contains “binary demographic variables”, I must have missed something here, aren’t these nine all latent factors?

**Response: You are right! We deleted this.**

>>Table 1 Footnote b: I may have missed that before, but this is the only time that you say that you used a second-order factor model, correct? You may want to clarify in Line 322-329 (and, if applicable, the other factors) whether the CFI reported there is from a two/three factor model with independent factors, with a second-order superfactor or maybe with correlated factors.

**Response: This is correct. We now included the following explanation in our instruments section:**

“When the three first-order factors are part of a higher-order factor, the resulting model is just-identified, which implies that the fit of the higher-order model should be identical to the first-order factor model. Because of this, we will use a second order model with the construct willingness to seek treatment as the higher-order construct and the aforementioned three factors as first order constructs in the main analyses.”

>>I also assume that the comment regarding treating the variables as continuous holds for the whole table and not just “Willingness to Seek Treatment”? If so, you may want to put it in the notes (although honestly, I also think you can omit that, it makes sense here anyway).

**Response: As all models in the resubmission are based on ML and not WLSMV, we have dropped the comment about treating the variable as continuous in the table.**

>>Table 1 Variable 7: You report earlier you used a three-factor model for this construct (even gave the names of the three factors), why and how (second order factor model maybe?) do you combine them here (but leave 3, 4, and 5 separated)?

**Response: You were correct in your assumption that we used a second-order factor, as can be seen in Fig. 1 and in the note accompanying table 1. We hope that the changes that we described in a previous comment regarding the CFA section for the construct (“**. Because of this, we will use a second order model with the construct willingness to seek treatment as the higher-order construct” etc.**) will help avoid this problem in the future.**

>>line 368-369: The beta is negative, why do you say it predicted positively?

**Response: The way that the WHO wellbeing items (e.g., “I have felt cheerful and in good spirits”) were scored on soscisurvey, negative scores indicate lesser well-being, i.e., (1) all the time, (2) most of the time, etc. Hence, our interpretation of that effect was correct. Nevertheless, in the original paper and pencil format of the scale, the scoring is supposed to be (5) all the time, (4) most of the time, etc. We are now including the inverted scores in our model to avoid confusion. Thank you for pointing this out to us!**

>>line 369-370: A null result in NHST does not imply that the variables were unrelated (see remark regarding stars above, it seems you may have been affected by this).

**Response: That is of course correct. We changed it to “not significantly related”. Upon re-reading the manuscript, we noted several other instances where it is implied that NHST null effects implicates the absence of an effect, which were also changed.**

>>line 369-370: I may miss that, but what variable do you think would be suppressing the correlation? Are you investigating this further or is this more of a side remark (considering that you then remove the predictor, I guess the latter, but I am a little confused here what you are aiming at).

**Response: We** have included a longer discussion of this in response to a remark from Reviewer 3. The section now reads as follows: “Psychological well-being positively predicted attitudes toward seeking treatment in the pre-registered SEM model (β = -.36, [-.33, -.05]). This indicates a suppression effect, as psychological wellbeing was not statistically related to attitudes toward seeking treatment in bivariate analyses (r = -.05, see Table 1). It was our initial intention to include the WHO-5 alongside the BSI-18 in order to include in our model constructs that are not solely from a deficit/disorder perspective. In hindsight, the strong conceptual overlap of these two measures and constructs was likely foreseeable as a potential problem and likely represents a theoretical error in model building on the part of the authors. It was therefore decided to remove psychological well-being from the model to increase interpretability and to account for the strong conceptual overlap between psychological wellbeing and psychological distress.” We hope that this explanation clarifies our line of thinking with regards to the suppression effect and model adjustment.

>>Model 1 / Model 2: You state that Model 1 had a fit you didn’t find satisfactory, Model 2 seems to have almost exactly the same fit (the CFI is very close, and it is not a very good fit index for such a model anyway since the independent model doesn’t make much sense as a lower cut-off, and the RMSEA is exactly identical). Do you feel Model 2 is an improvement? If so, I’m not sure I understand the reason.

**Response: The new manuscript now contains a more detailed explanation on why we decided to remove psychological wellbeing and anticipated stigma (please see our previous response and our response to Reviewer 3’s concerns).**

>>Table 2: Considering that both fit methods ended up with qualitatively mostly the same results, ML is more conservative in the results, ML is the one better grounded in theory, and the WLSMV fit method gave you convergence trouble with the “Willingness to seek treatment” variable so that you had to resort to Openess only (which I think is not the same thing substantially), I suggest to remove the WLSMV fit method completely from the article.

**Response: We have removed the WLSMV fit method, as explained in a response to an earlier comment.**

>>line 430-431: I do see why “Whenever I think about being an MAP, I feel depressed” and the depression subscale is correlated for MAP, this is a very hard theoretical reason to respecify the model from my point of view, contrary to your statement. It is legitimate to keep the model based on a spuriousity argument, I suppose, but I’d suggest to change the reasoning here.

**Response: The manuscript text was changed as follows: “We attempted two model specifications to account for this problem, 1) conducting the analyses without item 4, 2) including residual covariances between the item and the Depression subscale, changing the model fit to χ2 = 2039.41, df = 1408, p < .001, CFI = .91, RMSEA = .04, [.04, .05] and χ2 = 2163.44, df = 1461, p < .001, CFI = .91, RMSEA = .04, [.04, .05], respectively. As the changes in model fit compared to Model 2 were minimal, neither specification was adopted. There was no theoretical legitimization to respecify the model further.”**

>>line 437: By “adequate” you mean from a substantial/theoretical point of view? I don’t share that opinion, for me the other two subscales do contribute in a meaningful way. But it’s an opinion thing, so if you want to report just openess, okay. I’d suggest to generally remove the factor then and just go with openess everywhere. As mentioned above, I more strongly suggest to use the ML estimator.

**Response: We are now using the ML estimator.**

>>Table S10: I’ve difficulties to follow the logic here: Firstly, am I right that the column header for Column 2 should go in the column itself (that is, that only the first page are factor loadings, then this column is reflecting regression slopes, and so on)?

**Response: Table S10 is deleted in the current version of the manuscript.**

>>Second, why do you report all fixed-to-zero intercepts? Wouldn’t it be enough to say in the note “all intercepts were fixed to zero as all variables were moved to zero mean”? Thirdly, why do you fix all residual variances, and how did you get at the numbers you fixed them to?

**Response: Following your previous suggestion, we have excluded the WLSMV models.**

>>line 446-448: From the CIs in Table 2 (assuming the lower and upper bounds are bounds of a CI, you may want to mention that in the table), I take it the ML estimator mostly also reported as-good-as-significant effects here, right? In a Bayesian expression of these results, the difference would still be minor. Also, I’m curious what the ML model would do if you only used openess instead of willingness – it’s really difficult to compare the two approaches on different variables. All in all, I also think from these result that you may want to take the WLSMV estimator out.

**Response: We have changed the cell to make it clear that we are referring to the lower and upper bounds of a CI. As we have removed the WLSMV models, your second concern is no longer relevant.**

>>line 498-499: I’m careful here. It is a fact that visiting a psychotherapist is a substantial risk for pedohebephilia patients. I’d say convincing them by good advertisement is not the way to go as long as we cannot ascertain that the risk is in fact in correspondence to this representation of therapy. Note that I do think the majority of psychotherapists are well-intentioned, warm and successful (as most pedohebephilic persons are), but we do need to keep in mind the danger that comes from the very few exceptions.

**Response: This corresponds to a concern voiced by Reviewer 1, which we have responded to above. Short answer: We agree and hope that the revised version offers a more balanced view on this.**

Minor Issues (to use or not as you think best):

>>line 99 following: I’m not sure I understand these three sentences, do you mean the following: “A general anticipation of negative reactions, e.g., fearing to be verbally attacked in the street, did not show a reliable relation to willingness to seek treatment. It is likely that the connection is more prominent when comparing anticipation of negative reactions from a psychotherapist when confining in them.” If not, I’m sorry; you may want to define briefly what you mean by “general anticipation” and “degree of specificity”. Am I right that you assume the relation did not show because of a floor effect (because you say before that pedohebephilic persons are generally not very afraid of being confronted in the public)?

**Response: No. Pedohebephilic people are unlikely to be attacked in the public (as a form of experienced stigma), but they might still expect to be attacked in public if they are found out (as a form of general anticipated stigma). Hence, they tend to report low levels of experienced stigma (because others do not know about the attraction) and higher levels of anticipated stigma (because if others find out, this is likely to lead to negative consequences). When we talk about specificity, we are referring to the degree of similarity between the context described in two items. For instance, we would expect a stronger correlation between “I have eaten Indian food at least once in the last three months” and “I like Indian food” than between “I have eaten Indian food at least once in the last three months” and “I like spicy food”.**

**We changed the section as follows: “Prior research did not detect a significant link between general anticipation of negative reactions by unspecified others and attitudes toward seeking treatment in pedohebephilic individuals (12). Yet, this stigma process was not assessed with reference to a potential therapist who would be the recipient of disclosure rather than a vague or general group. It is likely that a link between anticipating negative responses and attitudes toward seeking treatment will be identified if both concepts are assessed with a similar degree of specificity (27), such as when focusing on anticipated negative reactions from a potential therapist.”**

>>line 115 “..., with one study...” -> “... in one study...” (phrasing it less generalizable from a single study).

**Response: The section has been changed as follows: “Yet, in one study the opposite effect appears to hold for LGB individuals, with participants who report a more negative view of their LGB identity reporting greater willingness to seek treatment (28)”.**

>>line 117 “This indicates...”: Seems a little strong for me to conclude this from one study looking specifically at LGB? Maybe you can rephrase it as a new hypothesis, “This makes us speculate that...” or “This leads to the possible speculation that...”

**Response: The section now reads “This leads to the speculation that stigmas other than mental illness stigma, internalized stigma could improve attitudes toward seeking treatment as an opportunity to discuss such negative feelings in a supportive environment.”**

>>line 120 “...to want treatment..” -> “..wanting treatment...”
>>line 127 “...approximately half...” -> “...approximately half of the sample...”

**Response: Both fixed.**

>>line 96 and 110: You may want to synchronize both headers (e.g., “[dimension] Stigma and Treatment Seeking” in both. Since you identify three dimensions before, one is kind of missing “experienced stigma”. I understand you say before that experienced stigma is rare in this community compared to other stigmata, but it still exists for mostly all pedohebephilic people (in rare but memorable ways), and it seems reasonable to assume that it does have an effect, even if more difficult to measure.

**Response: We changed the heading to “internalized stigma and treatment seeking”. No other changes were made.**

>>below line 66: I would suggest to give an overview of possible mechanisms about the problem of the interaction to lead readers in, e.g.: “The interaction between stigma and willingness to seek therapy might go in both directions, (1) people that fear therapy will not be effective or even hurtful because of stigma anticipated from the therapist or internalized stigma will be pushed away from seeking therapy, while (2) people how anticipate, experience or internalize stigma may suffer from that fact and see an increased need for therapy for themselves. Both mechanisms may also be active at the same time and cancel out effects. The literature reflects this in reports that may seem contradictory.”

**Response: We have included this approach in the second half of the introduction, which has been changed to: “In line with previous literature reviews demonstrating a link between stigma and negative attitudes toward seeking treatment (8,9), stigma related stressors may work to deter pedohebephilic individuals from seeking treatment (10,11). Yet, quantitative research has either failed to find a link between stigma and attitudes toward seeking treatment (12,13) or found that stigma is linked to a more positive attitudes toward seeking treatment in this population (14). We assume that the links between different stigma processes and willingness to seek therapy might go in opposite directions, (1) anticipating stigma from the therapist or having internalized stigma may push people away from seeking therapy, while (2) anticipating or internalizing stigma may increase the need for treatment because of the distressful nature of these experiences. Given the importance of reducing barriers to accessing mental health services, the present research aims to extend our understanding of the complex ways in which stigma relates to attitudes toward seeking treatment among pedohebephilic individuals.”**

>>line 146, very minor: It is no longer necessarily assumed true that cross-sectional data allows no causal inference, but it is of course correct that the current data does not. “prediction” is not per se linked to “causal” either.

**Response: We assumed that causality cannot be inferred when data are both cross-section (meaning that no temporal order can be established) and observational (meaning that no intervention is taking place). We wrote that “causality cannot be inferred because of the cross-sectional and observational nature of our data.”**

>>line 148: You may want to lead with “We hypothesize the following interactions:”

**Response: Done.**

>>line 201: “S1” -> “S4”

>>supplement Table S4 notes: “UM” doesn’t appear as header, I guess you mean “UDM”

**Response: all corrected.**

>>supplement Table S6 notes: “... a covariance...” -> “...an additional covariance of residuals...” (covariances exist between all items via the factor).

**Response: Upon re-thinking our modeling approach, we found decided not to include the additional covariance in the model to guard against spurious findings.**

>>line 217 “S2” -> “S7”

**Response: corrected.**

>>line 234 following: You may want to indicate more precisely how you compute the scores.

**Response: The section was changed as follows: “We used difference scores to determine 1) a Pedohebephilic Index (highest reported level sexual attraction to prepubescent or pubescent boys or girls minus highest reported level of sexual attraction to adult men or women), 2) a Male Pedohebephilic Index (highest reported level of sexual attraction to prepubescent or pubescent boys minus maximum level of sexual attraction to prepubescent or pubescent girls), and 3) a Pedophilic Index (maximum level of sexual attraction to prepubescent boys or girls minus maximum level of sexual attraction to pubescent boys or girls).”**

>>line 279 “continues” -> “continuous”
>>line 281 “... which may be...” -> “...which are...”

**Response: all corrected.**

>>Table 1 stars: I know it’s common, but among experts the stars are really frowned at, since they over-interpret post-fact NHST results and may even suggest that a lack of stars indicates a negligible correlation. My suggestion would be to mark (e.g., by making them bold) correlations you consider important substantively (e.g., that we get high correlations between variables 2, 3, 4, and 5 is not really surprising considering they come from very related constructs, three of them of the same scale).

**Response: no changes were made, but we appreciate that you shared this concern with us.**

>>Figure 1: The labels in the lower left three variables were cut off. Some variables are capitalized (“Psychological Distress”), other not (“Willingness to seek treatment”)

**Response: Thanks for catching this. We have corrected the labels and variable names.**

>>Figure 1: I’d suggest to exchange “Anticipated negative therapist behavior upon disclosure” and “Knowledge about psychotherapy” in the diagram to make the two mediation models more parallel.

**Response: Brilliant! Why hadn’t we thought of that before? The new figure looks much more elegant, and it is easier to spot the two mediation models.**

>>Line 373: I gather that the mediation effects are taken from Model 2? If so, you may want to clarify.

**Response: corrected.**

>>Table 2 Footnote a: I guess “Seeking Professional Help”, “Attitudes Toward Seeking Professional Help Scale” and “Willingness to seek treatment” all indicate the same thing here? If so, you may want to stay with the same name, if not (sorry), you may want to clarify.

**Response: This point is no longer relevant, as the WLSMV model has been deleted.**

>>line 423: “...247 were between |.10| and...” -> “...247 had absolute values between .10 and ...” (it doesn’t make sense to make absolute symbols around the .10 since it’s a positive value anyway).

**Response: Changed as follows: “Out of 1540 residual correlations, the absolute values (modulus) of 263 were between .10 and .20, 19 between .20 and .30, and none was larger than .30.”**

>>line 425: You may want to make sure that the line break is not within the number (-.10).

**Response: We trust that this will be fixed by the editing team later on, as the formatting of the article will be different in the proofs. Therefore, no changes were made.**

>>line 432 “Sensitivity tests...” -> “Sensitivity Tests...” (I think you want this to be a capitalized header since you capitalized elsewhere, right?)

**Response: According to journal guidelines, it should not be capitalized, and we have changed that throughout the whole manuscript.**

>>line 435 and other places: I think items should be capitalized; I’d be happy either way, but at the moment you do it differently at multiple places.

**Response: We have decided to not capitalize the scale names and have edited the manuscript to make sure this is consistent.**

>>line 436 “... Help because...” -> “...Help, because...”

**Response: The comma was added.**

>>line 461-462 “past research has found...to be unrelated” -> “past research has not found a relation between...” (no evidence of effect is not evidence for no effect)

**Response: True, but easy to overlook when writing a paper. We have changed the section accordingly: “This likely explains why past research has not detected significant links between general anticipated stigma (in non-specified social situations) and willingness to seek treatment** (Jahnke et al., 2015)**.”**

**###Reviewer #3:**

>>This is an interesting paper that has the potential to make a contribution to the field.

>>The introduction covers a lot of ground. It would be nice to see this restructured in a way that is focused less on the presentation of summaries of individual constructs, and more in line with an integrated argument that motivates the study. This is likely to be a restructuring rather than a re-writing of the introduction.

**Response:** We discussed this in the author team. In our opinion, the current structure works well: We introduce the problem (mixed findings on the link between stigma processes and help-seeking), introduce the individual constructs and how they relate to seeking treatment, discussing reasons why specific processes may show differential links to seeking treatment. Hence, no changes were made.

>>I appreciated the pre-registered nature of the project, which is rarely seen in this area of work. Setting the power analysis to 80% is the minimum that would be acceptable in published work and, while sufficient, does limit the rigor of the research. That said, the final sample is above this minimum threshold. Many of the measures used in the study were self-produced, which may limit the independent rigor and/or validity of the inferences drawn. I appreciated the efforts to present some validation of these measures, though this was not always convincing.

>>I was unclear as to why CFAs were conducted on the scales included in the study. Are there concerns about their factor structure? It felt like these analyses, if needed, could be presented in a supplement.

**Response: There were concerns in particular about the factor structure of all scales that were relatively new or developed for the present study. We have now removed all analyses for the established scales, as our preregistration only stated that the less established scales (knowledge about psychotherapy, internalized stigma, anticipated stigma, anticipated therapist reaction upon disclosure) should undergo CFA. This process is now explained more clearly in the data analysis plan and results section, where we now separate between stage 1 and stage 2 testing, e.g.,**

**“Stage 1. Assessing the factor structure of all new scales**

**Initially, we verified that the newly developed or less established scales functioned adequately on their own, based on RMSEA and CFI criteria. […]**

**Stage 2. Testing associations**

**After confirming adequate fit for the measurement models of the new scales, bivariate correlations were assessed based on the latent variables that emerged from conducting separate CFAs for all included scales. SEM model were used to test all variables as part of a larger, multifactorial model, with mediation effects. […] ”**

>> In terms of the SEM, I found the pre-registration document difficult to follow, and the set-up of the latent and observed variables felt a little convoluted. This made it difficult to assess whether the planned analytic approach was followed.

**Response: We have prepared a document where we listed all deviations from the preregistration alongside our reasons and an evaluation of the resulting risk for bias. We hope that this will make it easier for readers to evaluate our approach. This is referenced in the manuscript as follows, “Deviations from the preregistration are listed in S1 File.”**

The authors report a deviation from the hypothesized model, which led to them removing two variables which, at face value, feel incredibly important (and have been identified as such in prior work). I cannot see any justification for the removal of these variables other than the model fit of the SEM. It would be nice to see some discussion of this deviation from the planned analysis, as the removal of psychological wellbeing and anticipated stigma is inconsistent with prior work in this area.

**Response: Thank you for your constructive feedback regarding our model adjustments. We have reconsidered our decision to remove these two variables, and in the end, determined to only remove psychological wellbeing, while leaving anticipated stigma in the model.**

**We apologize for not communicating clearly enough why these variables were removed from the model. In retrospect, we may have anticipated that psychological well-being and psychological distress are so closely related conceptually, this would emerge as a problem when we attempted to substantiate our theoretical predictions in a structural model.** **It is likely that we had erred in our preregistered theoretical model and at this point we can merely say that this was perhaps an oversight and remove the well-being scale from the model. We have now re-structured the section and added a longer discussion to our manuscript to better convey the reasons for these adjustments in the results section.**

**“The CFI of the pre-registered model was below the threshold of .90, χ2 = 26447.24, df = 1743, p < .001, CFI = .89, RMSEA = .05, 95% CI [.04, .05]. Psychological well-being positively predicted attitudes toward seeking treatment in the pre-registered SEM model (β = -.36, [-.33, -.05]). This indicates a suppression effect, as psychological wellbeing was not statistically related to attitudes toward seeking treatment in bivariate analyses (r = -.05, see Table 1). It was our initial intention to include the WHO-5 alongside the BSI-18 in order to include in our model constructs that are not solely from a deficit/disorder perspective. In hindsight, the strong conceptual overlap of these two measures and constructs was likely foreseeable as a potential problem and likely represents a theoretical error in model building on the part of the authors. It was therefore decided to remove psychological well-being from the model to increase interpretability and to account for the strong conceptual overlap between psychological wellbeing and psychological distress.**

**After removing psychological well-being from the model, the fit for the modified SEM model (Model 2, see Fig 1 for an overview including standardized regression coefficients) was as follows, χ2 = 2170.61, df = 1462, p < .001, CFI = .905, RMSEA = .04, [.04, .05].”**

**Anticipated stigma was previously deleted from the model because we did not want to overburden the model with nonsignificant links that could not be substantiated in previous studies. Yet, when revisiting this decision, we agreed with the reviewer that this concept was too important to delete, especially since it was more distinct from other measures in the model (which was not the case for psychological wellbeing).**

**This led to the following changes in the results section:**

**“There were no significant indirect effects between internalized stigma or general anticipated stigma and attitudes toward seeking treatment via psychological distress. Of note, we discovered a small negative association between general anticipated stigma and psychological distress in both Model 1 and 2, even though the bivariate association between the two constructs was nonsignificant and in the opposite direction. This unexpected negative association may reflect a protective effect of general anticipated stigma, that only emerges when stripped of its overlap with internalized stigma. A comprehensive overview of all unstandardized model parameters for Model 1 and 2 can be found in the S5 – S6 Tables**.**”**

**From the discussion section:**

**“In line with expectations from the minority stress model (19) and previous research (25), internalized stigma was associated with more psychological distress. For general anticipated stigma, higher anticipated stigma was associated with less psychological distress and higher wellbeing in a SEM model. This finding hints at a potential mediating effect, as anticipated stigma might activate coping strategies to manage situations where the stigmatizing attribute might be discovered. We assume that this association, which was not detectable in bivariate analysis, could only emerge because internalized stigma absorbed much of the shared variance related to psychological distress. However, note that caution is also warranted when interpreting unexpected findings, and we want to stress that more research is needed to establish whether this result replicates.”**

>>The conclusions, as presented, make logical sense and I think that there is a role for these data in informing prevention service design. However, the inferences are only as strong as the measures used are valid. Given the self-produced nature of many of the measures used in the study, some caution is required in the interpretations. The authors also acknowledge the questionable psychometrics of some scales (e.g., the Attitudes Toward Seeking Professional Psychological Help Scale) and advise against their future use. In the case of the Attitudes Toward Seeking Professional Psychological Help Scale, the explicit statements about both past research (not finding adequate psychometrics) and future use (advising against this), casts doubt over the validity of what is a (or perhaps even the) fundamental variable in their model. This does limit the extent to which trust can be had in the results of this paper.

**Response:** We agree with the reviewer and hope that our critical discussion of this problem in the limitations section will help readers judge our findings: “For several scales, the proposed models did not fit our data as well as expected. It was possible to increase fit based on modifications, which were theoretically plausible, and, with the exception of the anticipated therapist reaction upon disclosure scale, minor. Nevertheless, we acknowledge that this practice can change the meaning of the scale in unforeseeable ways. The attitudes toward seeking professional psychological help scale specifically pertains to seeking professional help in response to emotional problems. Although previous studies show that such concerns are common among pedohebephilic people who seek treatment (65,70), other motives for treatment (such as seeking validation or coping with sexuality) are not covered explicitly by this scale. Moreover, future studies might consider assessing interest in counseling or support that do not require formal psychotherapeutic training and mental health professional status. Although we achieved a good fit for a three-factor higher-order structure for anticipated therapist reaction upon disclosure, concerns about potential overfitting warrant caution. Further research is needed to validate and possibly refine the factor structure of this scale to ensure its reliability and validity in future studies.”

The manuscript no longer includes a psychometric analysis of the more established scales, in line with our preregistration. Note that the three-factor model of the Attitudes Toward Seeking Professional Psychological Help Scale based on Picco et al. was also the one that was pre-registered.

>>Minor points:
>>- Consider removing statistical details from the abstract. These made it difficult to follow, and could obscure the potential importance of the work

**Response: The decision to include the statistical details was made with reference to APA journal article reporting standards, which prescribe the following: “Abstract: Report values for at least two global fit statistics, each from a different class, and include a brief statement about local fit (residuals). State whether the interpreted model (if any model is retained) is the originally specified model.” We did not follow the standards completely, as we thought that there was not enough space to include a discussion of local fit. Hence, we are faced with one side (represented by the APA guidelines) that requests even more statistical detail and another one (represented by the reviewer) who would prefer less. We are comfortable taking a middle-ground position and therefore, deleted only the regression coefficients, while leaving the fit indices in the abstract. But since we do not have strong feelings about the matter and believe that there are valid arguments for each side, we are happy to make additional changes, if the editor requests them.**

**Appelbaum, M., Cooper, H., Kline, R. B., Mayo-Wilson, E., Nezu, A. M., & Rao, S. M. (2018). Journal article reporting standards for quantitative research in psychology: The APA Publications and Communications Board task force report. *American Psychologist, 73*(1), 3.**
