# Supplementary material for: Stigma processes, psychological distress, and attitudes toward seeking treatment among pedohebephilic people
Source: PLoS One. 2024 Oct 24;19(10):e0312382. doi: 10.1371/journal.pone.0312382 (PMC11500907; doi:10.1371/journal.pone.0312382)
Supplement: Attachment — Submitted filename: letter to the reviewers.docx [file pone.0312382.s010.docx]

Dear Dr. Federici,

Thank you for allowing us to resubmit an edited version of our manuscript to PLOS ONE. We have addressed the remaining points and corrected a small mistake that has gone unnoticed in the review process so far.

Below, please find our responses to Reviewer 2’s remaining concerns.

Sincerely,

the authors

**####Response to reviewers**

####Journal Requirements:

>>Please review your reference list to ensure that it is complete and correct. If you have cited papers that have been retracted, please include the rationale for doing so in the manuscript text, or remove these references and replace them with relevant current references. Any changes to the reference list should be mentioned in the rebuttal letter that accompanies your revised manuscript. If you need to cite a retracted article, indicate the article’s retracted status in the References list and also include a citation and full reference for the retraction notice.

**Response**: We are not currently citing any retracted articles.

**####Additional Editor Comments:**

>>The reviewers felt that the manuscript was much improved in this revision. However, Reviewer #2 still raises some points that should be corrected to make the manuscript suitable for publication. This is a minor revision, another small effort.

**Response**: Thank you! We hope that all issues are addressed in our resubmission. In addition, we want to bring to your attention another mistake that we have now corrected related to an unintentional deviation from our preregistered stopping rule:

“Recruitment took place between 7th of January and 6th of May 2021. Hence, data collection was stopped exactly one month short of the 5 months that were pre-registered as the stopping rule due to an honest mistake on the part of the first author, not a decision based on the data or results.”

The mistake is also discussed in the supplementary materials on deviations from the preregistration.

**####Comments to the Author

Reviewer #1**

>>All my comments have been addressed. I could push back against certain interpretations, but I am satisfied with the article as it is.

**Response:** Thank you.

**####Reviewer #2:**

>>I would like to disclose that between the two review rounds, an event has occurred between one of the authors and me that could be perceived as creating a conflict of interest. I feel that I can review the article objectively nevertheless. I write this before starting to read the revision to make sure it can be factored in in case I’ll make critical remarks.

>>In my view, the authors did a great job to address my points, and, as far as I can judge, the points raised by the other two reviewers. I see still a need to make changes regarding issues 6 and 20 (which corresponds to a point raised by Reviewer 1) below, and I feel a little at a loss what the authors did regarding issue 16. Regarding all others, I either fully agree with the authors or have just some minor nagging. With these very few changes, I feel the article could make a great contribution to the field.

**Response:** Thank you. We will make sure to pay particular attention to issues 6, 20, and 16.

>>Reviewer 1 page 92 bottom of the reply letter, related to major issue 20 below: I would like to make an additional suggestion regarding this discussion with Reviewer 1: I see a possible problem by dividing therapists (ourselves as well as in the view of MAPs) into ‘good’ and ‘bad’ therapists, which is probably not better than dividing MAPs in similar categories. In my view, the very wide majority of therapists has best intentions, it’s just that they are human, too, with possibly difficult pasts. So I’d suggest to add that a campaign targeted at pedehebephilic persons should include suggestions how to communicate the situation to the therapist in a way that he or she can cope best and provide support most successfully.

**Response:** That’s a good idea! We have added this to our discussion: “To mitigate these risks, an intervention could provide the clients with strategies to describe their sexual interest in ways that increase their chances of receiving the support that they need, for instance by communicating that having sexual interests in children and committing sexual offenses are not the same. However, such an intervention component would have to designed carefully, as clients who are at risk of committing sexual offenses should not be encouraged to conceal or downplay risk-relevant factors in treatment. Furthermore, potential clients should be encouraged to seek treatment with therapists that are specialized in working with this client group, if possible.”

>>Regarding all issues including the WLSMV estimator, I think the authors did a great job in explaining why they removed it.

>>Re major issue 1 (original line 217), the new version is great. Very minor, the scale ranging from 1 to 101 would be a 101-point scale, not a 100-point scale; one of the two numbers seems to be in error.

**Response:** We fixed the section in our paper, where we now correctly state that we used a 101-point scale. Thanks for catching this mistake.

>>Re major issue 2 (297), I understand the reasoning and agree. You should add the missing treatment explicitly, I.e., I suggest “...ML estimation. The preregistered...” -> “... ML estimation. Using listwise deletion, the preregistered...”.

**Response:** Done.

>>Re major issue 3 (316), the authors in my view removed the problems successfully.

**Response:** We’re glad to hear that.

>>Re major issue 4 (factor scales strategies): I like the changes. Minors: “...scales function adequately...” -> “...scales worked adequately well...”, “...above .80 and...” -> “... above .80, and...”, “...of CFI = .90., which...” -> “... of CFI = .90, which...” (a dot too many), “...indicated that...” -> “... we used as a threshold to conclude that...”

**Response:** Done.

>>Re major issue 5 (line 321): I see the point is obsolete now.

>>Re major issue 6 and 7 (Table 1): I still feel that it seems odd to go to average scores here when you put so much work previously into setting up a good factor model, and that factor models (as I read the weights) fairly strongly says that average scores are not adequate. Sorry if I state the obvious here, but just to be sure we’re on the same page, an average score represents a factor score under the assumptions that all loadings are equal. This is why, given the unequal loadings in your factor model, it seems contradictory in itself suddenly reporting the average score here. I feel your aim of providing the reader with an idea about the central tendency might be better served by reporting the factor scores (lavaan produces those for you, I think; if not, Onyx does). It’s still bivariate analysis even if both variates are latent, so either way, a full SEM shouldn’t violate the preregistration (and preregistration in general never should keep us from thinking about improvements, that would be a negative outcome of preregistration).

**Response:** We understand the reviewer’s misgivings with regards to mentioning any average composite scores in a SEM paper that acknowledges and shows that the items do not contribute equally to the latent score. The reviewer is correct that we could extract factor scores in lavaan (using the lavPredict function). We have in fact already done that to calculate the bivariate associations in Table 1, which are based on the latent scores from the CFA. However, if we were to average these latent scores, the means would come out as zero, because they are standardized. Hence, this would defeat the purpose of giving readers a sense of central tendency. Judging from the high correlation between the average composite scores and the latent scores, we are confident that composite scores convey a sufficient representation of the underlying constructs: r = .97 for Attitudes toward seeking treatment, r = .99 for psychological well-being, r = .96 for Psychological distress, r = .99 for Knowledge about psychotherapy, r =.99 for Anticipated negative therapist behavior upon disclosure, r =1.00 for general anticipated stigma, r = .99 for internalized stigma. While purists may disagree, we believe that in this instance, average composite scores are the best way of conveying how participants scored on each of the variables. Therefore, we wish to maintain the use of average composite scores as a pragmatic solution in this particular situation.

>>Re major issue 8 and 9 (Table 1): The solution the authors chose seems reasonable to me.

>>Re major issue 10 (368): Also seems a good solution sufficient to me.

>>Re major issue 11 (369): Okay; not ideal, I’d suggest to go with a Bayesian interpretation here, but at least it’s formally correct now, even though I fear the null result may still be miss-understood as confirming the null by most readers.

**Response:** The primary focus of this section is to explore the possibility of a suppression effect. Given that the SEM model shows not only a non-significant effect but also a change in direction (from positive to negative) compared to the bivariate correlation, we interpret this as indicative of a suppression effect. While we understand your concern about readers misinterpreting a null result as evidence for a lack of effect, we also need to be concerned about readers misinterpreting a negative (nonsignificant) finding as evidence for an effect in the opposite direction.

>>Re major issue 12 and 13 (369): This seems a more than thorough solution to the problem.

>>Re major issue 14 (Table 2): Obsolete now.

>>Re major issue 15 (430): Seems good enough to me.

>>Re major issue 16 (437): Okay about the ML estimator of course, but that was only a side remark by me, the main point was about reducing to the openess factor. Sorry if I missed that in your reply in case it was included somewhere above, but how did you change the treatment of the factors?

**Response:** We can see why this response was confusing for you, sorry for not having been clearer. The reason we used the openness scale in a previous version of the paper was because the model did not converge previously when we used the full scale for the WLSMV estimator. This was never a problem for ML, so we had no reason to switch to the openness scale in the first place. In other words, this point is obsolete now.

>>Re major issue 17 and 18(Table S10): obsolete!

>>Re major issue 19 (446): Also obsolete mostly, the minor is corrected.

>>Re major issue 20 (498): See remark regarding Reviewer 1’s concern about the same point; I like what you did, but I’d suggest to add the component of “helping the therapist along”.

**Response:** The current manuscript now includes a sentence that stresses this component, as described previously. We hope that these changes will address your remaining concerns.

>>As I thinking this over again, as much as I hate to say this: I do suggest that you add explicitly “In very rare case, visiting a psychotherapist can result in a life-threatening situation.” It’s a rare exception and it shouldn’t keep MAPs from seeing psychotherapists when all is said and done, but so is CSA, and it shouldn’t keep children from interacting with MAPs, but that doesn’t mean we should not be honest about the existence of the risk. You may of course add a big “but” afterwards!

**Response:** Thank you for this suggestion. While we acknowledge that negative treatment experiences can, in rare cases, lead to life-threatening situations (in the presence of additional risk factors like substance abuse, depression, social isolation, or impulsivity), our focus is on presenting the most relevant information for more common cases encountered in practice, as informed by our previous review of the literature (Chronos et al., 2024). However, the present version of the manuscript is more explicit about the risks of disclosure, mentioning that many clients face “rejection, hostility, or abandonment” when disclosing their sexual attraction to children. We hope that this will address the reviewer’s concerns and strengthen the manuscript without delving into more extreme scenarios.

Chronos, A., Jahnke, S., & Blagden, N. (2024). The treatment needs and experiences of pedohebephiles: a systematic review. Archives of Sexual Behavior, 1-18.

>>Re minor (146 in the original manuscript): Your assumption was considered general knowledge until very recently. However, newest literature suggests that contrary to what we believed before, it may in fact be possible to deduce causality from cross-sectional observational data when not all variables involved are normally distributed. But this is cutting edge, and your data is normal, so unless you’re really strict about not saying anything that is very technically wrong, just leaving this as it is is just fine in my view.

Re author replies to Reviewer 3, I have no comments to add.

**Response:** We hope the current manuscript addresses the remaining points and would like to thank the reviewer for their assistance.

####Reviewer #3:

 The manuscript is much improved and the authors have done a good job in addressing most reviewer comments.

**Response:** Thank you for your assistance in improving the manuscript.
